# Shear wave elastography combined with the thyroid imaging reporting and data system for malignancy risk stratification in thyroid nodules

**DOI:** 10.18632/oncotarget.15018

**Published:** 2017-02-02

**Authors:** Zhao Liu, Hui Jing, Xue Han, Hua Shao, Yi-Xin Sun, Qiu-Cheng Wang, Wen Cheng

**Affiliations:** ^1^ Department of Medical Ultrasound, Harbin Medical University Cancer Hospital, Harbin, Heilongjiang, China

**Keywords:** thyroid nodules, shear wave elastography (SWE), thyroid imaging reporting and data system (TI-RADS), “tandem” and “parallel”, diagnostic performance

## Abstract

To retrospectively evaluate the diagnostic performance of shear wave elastography (SWE) and thyroid imaging reporting and data system (TI-RADS) in differentiating malignant and benign thyroid nodules. A total of 313 thyroid nodules in 227 patients were included. All thyroid nodules were underwent SWE and TI-RADS before fine needle aspiration biopsy and/or surgery. SWE elasticity indices of the maximum (E_max_), mean (E_mean_), minimum (E_min_) and elastic ratio (ER) in thyroid nodules were measured. Nodules with solid component, marked hypoechogenicity, poorly defined margins, micro-calcifications, and a taller-than-wide shape were classified as suspicious at gray-scale ultrasonography. The level of TI-RADS was determined according to the number of suspicious ultrasonography features. The combined methods of SWE and TI-RADS in thyroid nodules were calculated. In the 313 nodules, 194 were malignant, and 119 were benign. SWE and TI-RADS were significantly higher in malignant nodules than benign nodules (*P* < 0.001). The most accurate SWE cut-off value, 51.95 kPa for E_max_, achieved a sensitivity of 81.44% and a specificity of 83.19% for discriminating malignant nodules from benign nodules. There are two methods in combination with SWE and TI-RADS. The one is “tandem” method, which has a higher specificity (95.80%), positive likelihood ratio (18.16) and positive predictive value (96.73%). The other one is “parallel” method, which shows sensitivity (94.85%), negative likelihood ratio (0.07) and negative predictive value (90.00%).We believe that the methods could be used as a simple tool to stratify the risk of thyroid nodules accurately.

## IFIGNTRODUCTION

Thyroid nodular disease (TND) is one of the most widespread endocrine disorders. In recent years, the incidence of TND has gradually increased, and approximately 5% to 15% of them are malignant nodules [1]. Although conventional ultrasonography (US) has become the preferred imaging method for diagnosing thyroid diseases, its key limitation includes poor differentiation of benign from malignant nodules. TND are found in up to 67% of adults by US [2]. However, fewer than 5.0-6.5% of incidentally discovered TND are malignant [3]. Thyroid nodule ultrasound characterization performed by experienced clinicians allows the selection of tumors to be punctured and guides fine needle aspiration (FNA) biopsy. FNA plays an important role in differentiating TND because of its high sensitivity and specificity [4, 5]. However, FNA biopsy shows numerous weaknesses, FNA is an invasive method and may have a false-negative rate or a false-positive rate [6, 7] and reveals high nondiagnostic (10-15%) or indeterminate (10-20%) possibility [8].

Conventional US is recommended as the initial examination for all patients with known or suspicious thyroid nodular disease [9–11]. In breast imaging, the Breast Imaging Reporting and Data System (BI-RADS) is widely used to assess the probability of malignancy [12]. Based on BI-RADS, the terminology of Thyroid Imaging Reporting and Data System (TI-RADS) was first used by Horvath *et al*. [13], which described 10 US features of thyroid nodules. Subsequently, Park *et al*. [14] proposed an equation for predicting the probability of malignancy in thyroid nodules based on 12 US features. The Korean scholar Kwak *et al*. [15] set up a relatively simple TI-RADS classification standard according to the 5 malignant US features of thyroid nodules. Recently, Under the auspices of the American College of Radiology (ACR), a committee was organized to develop TI-RADS which consisted of six categories [16]. However, at present, for TND, a unified standard still does not exist.

Shear Wave Elastography (SWE) is a new, promising, but still not widely available technique. It is thought to be more objective, reliable and reproducible than older variants of elastography. [17–23] Previous report have suggested that SWE may add a new dimension to ultrasound evaluation of TND [17]. In SWE, shear wave emission is induced by a focused ultrasonic beam. Based on the received signals, the elasticity of the tissue is assessed in real-time and may be estimated both qualitatively and quantitatively. To date, few studies have reported the diagnostic performance of SWE in differentiating thyroid nodules [17–23].

Therefore, the main aim of this study was to evaluate the diagnostic performances of SWE and the TI-RADS score in differentiating benign and malignant thyroid nodules, using cytologic or histopathological analysis as the reference standard.

## RESULTS

### Demographic and pathologic characteristics

The final status of the 313 thyroid nodules was benign in 119 (38.0%) and malignant in 194 (62.0%). One hundred ninety-one malignant lesions were confirmed as papillary thyroid carcinomas, and 3 cases were medullary thyroid carcinomas based on surgical specimens. Most of the benign nodules were nodular goiters (*n* = 105), and 13 cases were adenoma except for one confirmed case of subacute thyroiditis. The basic characteristics of thyroid nodule is presented in Table 1.

**Table 1:** Basic characteristics of 227 patients who were confirmed by cytology or thyroid surgery

Characteristic	Benign (*n* = 119) *n* (%)	Malignant (*n* = 194) *n* (%)	Total	Chi-squared *t*-value*	*P* value
Gender			227		
Male	17 (42.5)	23 (57.5)	40	1.594	0.2067
Female	60 (32.1)	127 (67.9)	187		
Age	49.61±10.97	44.35±8.47	46.14±9.70	t=4.476*	< 0.0001
Nodule size(mm)	19.7±15.1	12.6±9.2	15.3±12.3	t=4.629*	< 0.0001
Solitary nodule	57	93	150	3.283	0.0700
Multiple nodules	20	57	77		
Location			313		
Left lobar	55	87	142	4.395	0.1111
Right lobar	64	100	164		
Isthmus	0	7	7		

### Diagnostic performance of SWE

E_max_, E_mean_, E_min_ and ER of SWE were significantly higher in malignant nodules than in benign nodules (*P* < 0.001) (Table 2). According to our data, the ROC curves of the four SWE parameters are shown in Figure 2A. The optimal cut-off values with respective AUC values are presented in Table 3. Compared with other SWE parameters, E_max_ with the optimal cut-off value set at 51.95 kPa had the highest AUC value (88.18%; 95% CI: 84.27 to 92.09), showing a diagnostic sensitivity, a specificity, a PLR, a Youden's index, a PPV and an NPV of 81.44%, 83.19%, 4.85, 64.63%, 88.76% and 73.33%, respectively (Table 3).

**Table 2:** Mean value and range using SWE of the thyroid nodules

Histopathology	E_max_* (kPa)	E_mean_*(kPa)	E_min_* (kPa)	ER*
Benign	41.3±14.8	25.5±10.8	14.2±8.4	1.25±0.39
Malignancy	73.0±35.7	39.3±17.1	19.2±11.7	1.82±0.56
Total	60.9±33.3	34.1±16.5	17.3±10.8	1.61±0.58

E_max_: the maximum elasticity index of the stiffest portion of the mass or surrounding tissue.

E_mean_: the mean elasticity index of the stiffest portion of the mass or surrounding tissue.

E_min_: the minimum elasticity index of the stiffest portion of the mass or surrounding tissue.

ER: the ratio of mean elasticity index of the lesion and parenchyma.

* Statistically significant difference of the SWE indices between malignant and benign lesions (*P* < 0.001).

**Figure 1:**
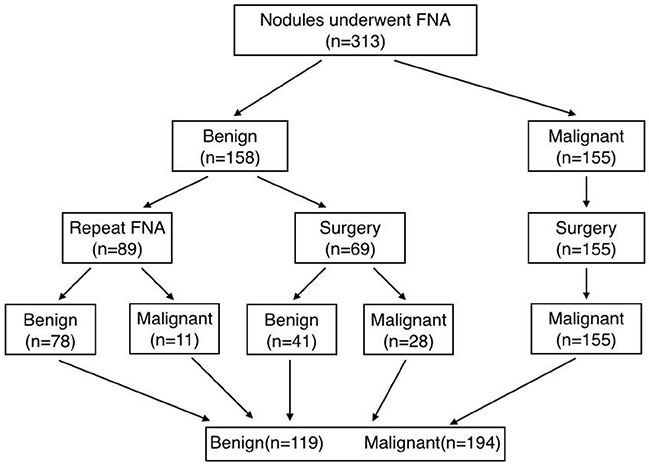
Flow chart of the study group FNA: fine-needle aspiration cytology.

**Figure 2:**
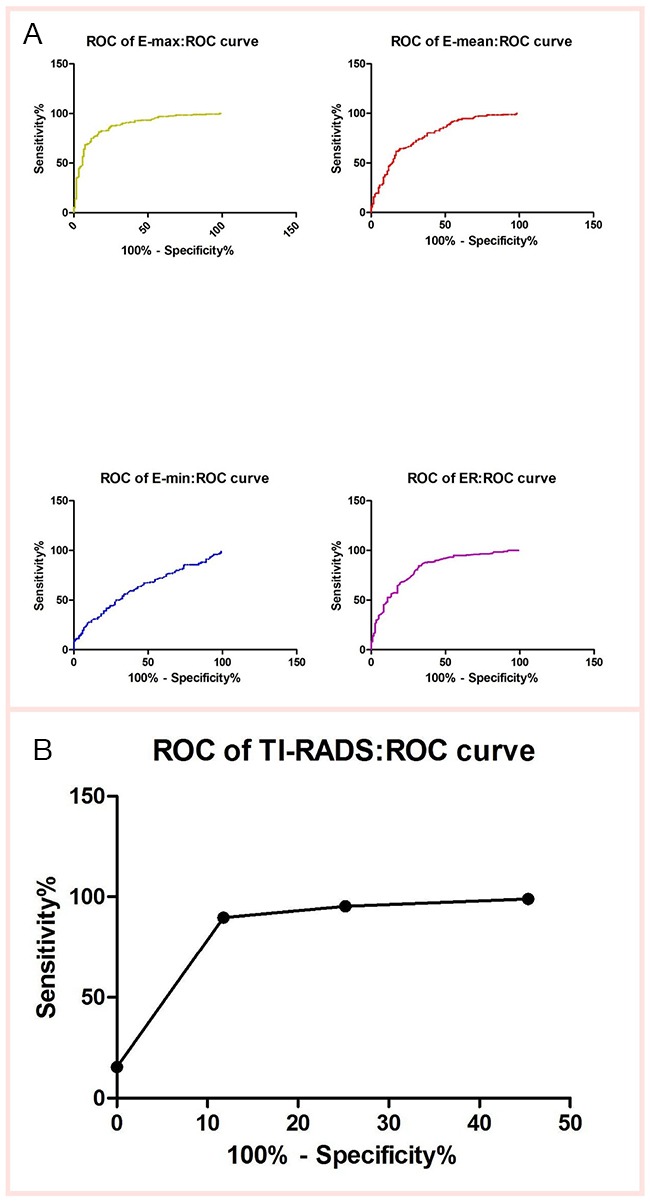
Receiver-operating characteristic (ROC) curves for the EIs of SWE and TI-RADS

**Table 3:** Diagnostic performance of elasticity indices for predicting malignancy on SWE

Elasticity indices (kPa)	AUC (%) (95% CI)	Cut-off values (kPa)	Sensitivity (%)	Specificity (%)	PLR	Youden's index (%)	PPV (%)	NPV (%)
_Ema_x	88.18 (84.27 to 92.09)	51.95	81.44	83.19	4.85	64.63	88.76	73.33
E_mean_	78.19 (72.92 to 83.46)	31.65	64.43	80.67	3.33	45.10	84.46	58.18
E_min_	62.50 (56.30 to 68.69)	16.45	55.67	66.39	1.66	22.06	72.97	47.88
ER	81.87 (77.03 to 86.71)	1.365	84.54	68.07	2.65	52.61	81.19	72.97

PLR, positive likelihood ratio; PPV, positive predictive value; NPV, negative predictive value.95% CI: 95% Confidence interval

### Diagnostic performance of conventional US and TI-RADS

Diagnostic performances based on a single conventional US feature are shown in Table 4. Micro-calcifications with the highest Youden's index (60.68%) was the conventional US feature most predictive of malignancy. The distribution of TI-RADS was cited as follows (Table 5). In this study, the percentages of malignant nodules are slightly superior to those of Kwak *et al*.'s [15]. According to our data, the ROC curves of the TI-RADS are shown in Figure 2B. The highest AUC value of TI-RADS with the optimal cut-off value set at TI-RADS 4c was 92.56% with a sensitivity of 89.69%, a specificity of 88.24%, a Youden's index value of 77.93%, a PPV of 92.55% and an NPV value of 84.00% (Table 6).

**Table 4:** Diagnostic performance of each conventional ultrasound characteristic

US features	Malignant (*n*=194)	Benign (*n*=119)	Sensitivity (%)	Specificity (%)	PLR	Youden's index (%)	PPV (%)	NPV (%)
Composition			95.36	48.74	1.860	44.10	75.20	86.57
Solid (n=246)	185	61						
Partial solid or Cystic (*n*=67)	9	58						
Echogenicity			93.30	59.66	2.313	52.96	79.04	84.52
Hypoechogenicity or Marked Hypoechogenicity (*n*=229)	181	48						
Hyperechogenicity or Isoechogenicity (*n*=84)	13	71						
Calcifications			85.05	75.63	3.490	60.68	85.05	75.63
Microcalcifications (*n*=194)	165	29						
Macrocalcifications or No calcifications (*n*=119)	29	90						
Margin			57.73	85.12	3.881	42.85	86.15	55.68
Microlobulated or irregular margins (*n*=130)	112	18						
Well defined (*n*=183)	82	101						
Shape			31.96	93.28	4.754	25.24	88.57	45.68
Taller than wide (*n*=70)	62	8						
Wide than Taller (*n*=243)	132	111						

PLR, positive likelihood ratio; NLR, negative likelihood ratio; PPV, positive predictive value; NPV, negative predictive value;

**Table 5:** Malignancy rate according to TI-RADS of 313 thyroid nodules surgically resected at a single center

TI-RADS categories	No. of cases (*n*)	Pathological results (*n*)		Malignancy rate^a^ (%)	Malignancy rate^b^ (%)
		Benign	Malignant		
3	67	65	2	3.0	2.0-2.8
4a	31	24	7	22.6	3.6-12.7
4b	27	16	11	40.7	6.8-37.8
4c	158	14	144	91.1	21.0-91.9
5	30	0	30	100	88.7-97.9
Total	313	119	194		

^a^ The rate of malignancy in this study; ^b^ The rate of malignancy in Kwak *et al*'s study.

**Table 6:** Comparison of the diagnostic performances of TI-RADS alone, SWE alone, “parallel” and “tandem” with histopathological/cytological results

Imaging method		Malignant (*n*=194)	Benign(*n*=119)	AUC (%)(95% CI)	Sensitivity (%)	Specificity (%)	PLR	NLR	Youden's index (%)	PPV (%)	NPV (%)
TI-RADS alone (4c+ as cut-off)	+	174	14	92.56 (89.26 to 95.86)	89.69	88.24	7.62	0.12	77.93	92.55	84.00
	-	20	105								
SWE alone (E_max_ =51.95kPa as cut-off)	+	158	20	88.18 (84.27 to 92.09)	81.44	83.19	4.85	0.22	64.63	88.76	73.33
	-	36	99								
Combined TI-RADS +SWE (parallel)	+	184	29	#	94.85	75.63	3.892	0.07	70.48	86.38	90.00
	-	10	90								
Combined TI-RADS +SWE (tandem)	+	148	5	#	76.29	95.80	18.16	0.25	72.09	96.73	71.25
	-	46	114								

AUC, areas under the ROC curve; PLR, positive likelihood ratio; NLR, negative likelihood ratio; PPV, positive predictive value; NPV, negative predictive value; 95% CI: 95% Confidence interval.

# It did not calculate the AUC of “parallel” and “tandem” in this study.

### Comparison of SWE and TI-RADS

Generally, the AUC of SWE parameters were slightly lower than those of TI-RADS (Table 6). Moreover, the values of TI-RADS were slightly superior to those of SWE in the sensitivity, specificity, PLR, Youden's index, PPV and NPV (Table 6).

### Combined application of SWE and TI-RADS

On the whole, the Youden's index value of the “tandem” (72.09%) was substantially same as that of the “parallel” (70.48%). Regarding the two cases used alone, the “tandem” has a higher specificity (95.80%), PLR (18.16) and PPV (96.73%), which were better than specificity (75.63%), PLR (3.892) and PPV (86.38%) of the “parallel”, and the “parallel” is more significant in terms of sensitivity (94.85%), NLR (0.07) and NPV (90.00%), which were superior to sensitivity (76.29%), NLR (0.25) and NPV (71.25%) of the “tandem” (Table 6).

**Figure 3:**
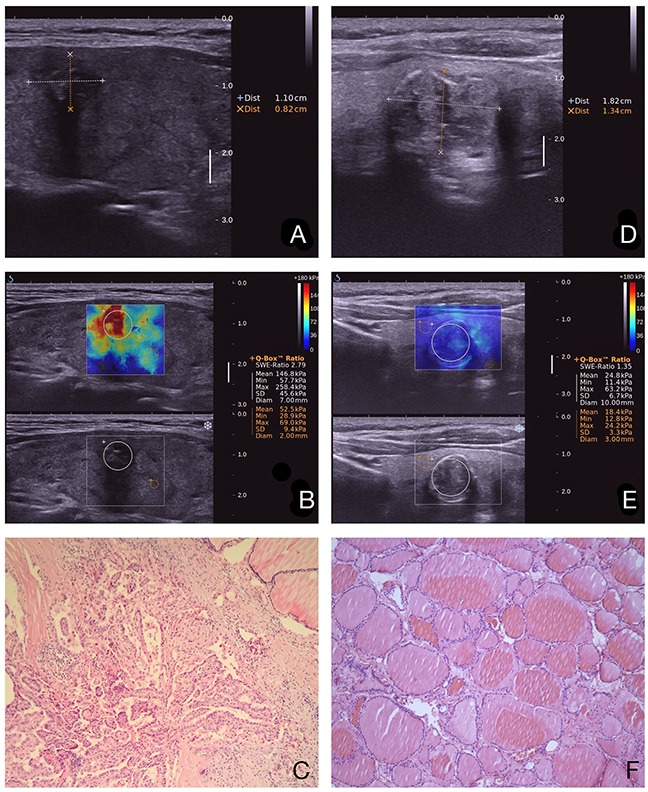
Images in a 64-year-old woman who underwent a routine checkup An 11-mm left thyroid solid nodule with marked hypoechogenicity, poorly defined margins, and micro-calcifications was found on gray-scale US, was classified as TI-RADS 4c **a**. The E_max_ value of SWE of the nodule was 258.4 kPa **b**. This thyroid nodule was diagnosed as papillary thyroid carcinoma after surgery. Pathological images (**c**. HE 10×10). Images in a 59-year-old woman who underwent a routine checkup. An 18-mm left thyroid nodule with macro-calcification was found on conventional US, was classified as TI-RADS 4a **d**. The E_max_ value of SWE of the nodule was 63.2 kPa **e**. The post-operational histopathology was nodule gotiers. Pathological images (**f**. HE 10×10).

Figure 3(a, b, c) showed typical malignant nodule correctly classified by SWE and TI-RADS. Ten of twenty-one nodules with macro-calcifications or eggshell calcifications (Figure 3d) were benign, and showed a false-positive result in SWE (Figure 3e, 3f).

## DISCUSSION

The great prevalence of TND makes the distinction between benign and malignant lesions a vital problem in endocrinology. Sonographic appearance is very helpful for the diagnosis and management of malignant and benign nodules [27–29]. SWE is a novel technique with high sensitivity and specificity in the evaluation of TND and can potentially reduce unnecessary fine-needle aspiration biopsies [30]. Liu *et al*. [31] demonstrated that SWE was helpful in predicting malignant thyroid nodules with comparable results. Consistent with previous studies, we also proved that the EIs of SWE had very significant differences in differentiating benign and malignant thyroid nodules (*P* < 0.001).

Generally, although Veyrieres *et al*. [18], Bhatia *et al*. [19], Sebag *et al*. [20] and Kim *et al*. [21], respectively, reported that a significantly higher EIs was noted in malignant nodules than in benign nodules’, the most accurate cut-off value of SWE has not been unified until now. The explanation for the cut-off value being different from the other studies may be due to the choice of different standards: we used the maximum Youden's index, while they used the best possible NPV [18] or a PPV of at least 80% [20]. We selected the best cut-off value in E_max_ ( = 51.95 kPa) when the Youden's index is maximum, with an AUC value of 88.18% and a sensitivity, a specificity, a PLR, a Youden's index value, a PPV and an NPV of 81.44%, 83.19%, 4.85, 64.63%, 88.76% and 73.33%, respectively.

There are some concerns in relation to potential limitations in SWE recordings that relate to issues such as arterial pulsation, calcifications and liquid content within individual nodules. A study [19] reported no difference in SWE indices between calcified and non-calcified lesions. However, nodules associated with macro-calcifications or egg shell calcifications showing a high false-positive rate for malignancy on SWE were reported by Sebag *et al*. [20], as same as our situation (Figure 3e, 3f). Moreover, lymphocytic infiltration and fibrosis, which modify thyroidal structure, may result in a change in thyroidal stiffness [23]. These could be limitations of elastographic US for the diagnosis of TND.

To avoid unnecessary surgical resection or biopsy in thyroid nodules, a high sensitivity and a high NPV of ultrasound screening were required for surgical decision making [32]. The most predictive of malignant US feature was micro-calcifications with the highest sum of the sensitivity and specificity. As one of main characteristics of papillary thyroid carcinoma, psammoma bodies may be the pathological basis of micro-calcifications [31]. However, no single US feature carries a sufficiently high accuracy in distinguishing between benign and malignant thyroid lesions, but the combination of multiple characteristics greatly increases the sensitivity and specificity [27]. Nondiagnostic thyroid nodules without suspicious US features or those with one suspicious US feature can be followed up with US, but nondiagnostic nodules with two or more suspicious features should undergo repeat US-guided FNA [33]. Horvath *et al*. [13] developed the TI-RADS to stratify thyroid cancer risk for clinical practice (sensitivity, 88%; NPV, 88%), and Russ *et al*. [25] demonstrated that the TI-RADS has a high sensitivity (95.7%) and NPV (99.7%) for diagnosing thyroid carcinoma. More recently, Kwak *et al*. established the classifications in the TI-RADS system [15], which we used in the research, and provided ultrasonographers with more information to classify benign and malignant nodules. According to our results, the pooled sensitivity and NPV of TI-RADS were 89.69% and 84.00%.

To our knowledge, one important finding in our study is that the combination of SWE and TI-RADS, to a certain extent, increases the diagnostic performance in differentiating benign and malignant thyroid nodules. When we used the SWE and TI-RADS in “parallel”, we obtained a higher sensitivity (94.85%), NLR (0.07) and NPV (90.00%) compared with the two methods used alone. This result suggests that the “parallel” approach can more effectively avoid unnecessary surgery and biopsy in TND. Particularly, when SWE and TI-RADS were simultaneously negative, namely the “parallel” method was negative, we can recommend the patients to be regularly follow-up every six months. In addition, the “tandem” method of SWE and TI-RADS is better than the two methods used alone, with a specificity of 95.80%, a PLR of 18.16 and a PPV of 96.73%. In other words, if the “tandem” method were positive—that is, SWE and TI-RADS were simultaneously positive—we should consider that thyroid nodules were possibly high-risk of malignancy, and suggest they undergo FNA and/or surgery in time.

It is worth mentioning that the increment of sensitivity is from 89.69% of TI-RADS alone to 94.85% with the combination TI-RADS and SWE in “parallel” with a lower specificity of 75.63% (TI-RADS alone had specificity of 88.24%). Mainly because the statistical way in which we chose, including “parallel” and “tandem”, led to the situation in the “Materials and methods”. In addition, we did not calculate AUC of “parallel” and “tandem” (Table 5), because we were not aimed to compare which of the two methods is better, but to play their own advantages to increase the diagnostic performance in differentiating benign and malignant thyroid nodules. Therefore, in our opinion, SWE and TI-RADS can form a complementary relationship in term of advantages. TI-RADS can compensate for the limitations of SWE that may be disturbed by macro-calcifications and carotid artery. Moreover, SWE can compensate for TI-RADS, which can be influenced by operator-dependence with inevitable observer variability. Especially, we suggest doing SWE for TI-RADS 3-4a nodules as a complementary tool, other than did the diagnosis separately.

There are several potential limitations of our study. First, The major bias is that this is a retrospective study based on results deriving by the performances of 7 operators. we only considered the nodules with all of the data based on the same and specific protocol to minimize this problem that reflected the variation among seven radiologists in clinical practice. Second, there were only 313 nodules from 227 patients included in our study. Moreover, almost all of the malignant nodules (191/194) were papillary carcinoma, while only 3 nodules were medullary carcinoma, and most of the benign nodules were nodular goiters. Other pathological types were not included, such as follicular thyroid carcinoma and anaplastic thyroid carcinoma. The number of cases is relatively small and the sampling/selection bias appears significantly high. Third, this study was based on a single specialized thyroid unit experience, so the data need to be tested by prospective multicenter and nonspecialized members. Fourth, there was an unusually high proportion of malignant nodules in our study group, a reflection of their referral center status. Finally, selection bias may exist because patients included in our study were scheduled for US-guided FNA for known thyroid nodules with suspicious US features or the largest one of multiple thyroid nodules that did not have any suspicious US feature. These may decrease the diagnostic performance on TI-RADS, causing false-negative cytologic results.

Both SWE and TI-RADS could be effectively performed to differentiate between benign and malignant thyroid nodules. Furthermore, we believe that the combined SWE and TI-RADS score could be used as a simple tool to stratify the risk of thyroid nodules accurately and may help to guide clinicians when making surgical decisions.

## MATERIALS AND METHODS

The retrospective study was performed in accordance with the ethical guidelines of the Declaration of Helsinki that was created by the World Medical Association [24], and was approved by the local Ethics Committee of Harbin Medical University Cancer Hospital. All of the participants were informed of the details and gave their written informed consent.

### Patients

From December 2013 to August 2014, 380 thyroid nodules were imaged at conventional US and SWE, by one of seven radiologists with 2 years of elastography experience and 10 years of thyroid US experience, who used the same protocol. Of these, 67 completely cystic nodules were excluded, 246 completely solid nodules and 67 partially solid nodules were included. Thyroid nodules that met the following criteria were included: 1) benign or malignant results at cytologic evaluation, 2) thyroid surgery was performed after obtaining cytologic results suspicious for papillary thyroid carcinoma or indeterminate results, such as follicular or Hürthle cell neoplasm, or when the nodule was benign but causing compressive or clinical symptoms or the patient's individual request, or 3) benign or malignant results at follow-up US-guided FNA or thyroid surgery after cytologic results of inadequate specimen (Figure 1). The exclusion criteria were as follows: 1) only diffuse thyroid disease was observed; 2) a cystic nodule comprising a completely liquid component; 3) a history of radiation therapy of the head and neck region.

Finally, 227 patients with 313 nodules scheduled were enrolled in this study (Table 1). 227 patients (46.14 ± 9.70 years; range, 12 - 73 years) were included in this study; patients included 187 women (45.60 ± 9.48 years; range, 12 - 73 years) and 40 men (48.65 ± 10.41 years; range, 28 - 72 years).

Two hundred twenty-seven patients underwent US-guided FNA for 313 thyroid nodules. One patient underwent US-guided FNA for four nodules, 7 patients underwent US-guided FNA for three nodules, 69 patients underwent US-guided FNA for two nodules and 150 patients underwent US-guided FNA for one nodule, respectively. 224 nodules in 159 patients performed thyroid surgery after FNA.

### Conventional US and TI-RADS

All conventional US and SWE scans were performed using a real-time US device (Aixplorer; SuperSonic Imagine, Aix en Provence, France) equipped with 4-15 MHz liner transducer. Conventional US was performed by one of seven radiologists with 10 years or more experience in thyroid imaging. Gray-scale US features of the detected thyroid nodules were recorded in the radiology reports for final assessment.

According to a previous study by Kwak *et al*. [15], the following US features showed a significant association with malignancy: a solid component, hypoechogenicity or marked hypoechogenicity, microlobulated or irregular margins, micro-calcifications, and a taller-than-wide shape. As the number of suspicious US features increased, the fitted probability and risk of malignancy also increased. With these findings, Kwak et al created TI-RADS category 3 (no suspicious US features), 4a (one suspicious US feature), 4b (two suspicious US features), 4c (three or four suspicious US features), and 5 (five suspicious US features). Kwak *et al*. suggest that macro-calcifications (without associated micro-calcifications) are not a risk factor for malignancy. In this study, all thyroid nodules were evaluated with TI-RADS category by Kwak *et al* [15].

### SWE

After conventional US, SWE was routinely performed by the same radiologists. SWE was performed in thyroid nodules detected at gray-scale US and targeted for US-guided FNA by using the same US machine and probe. All SWE images were obtained. Prior to performing SWE, each of seven radiologists had 2 years of elastography experience and 2 months of experience with the machine and weekly thyroid imaging conferences regarding elastography images.

According to depth of thyroid nodule from skin surface, we regulated frequency of liner transducer in order to show the images more clearly. After identification of the target lesion, the transducer was kept in a stable position without pressure about 3 seconds, perpendicularly to better minimize the compression artifact, and the SWE mode was implemented over the conventional US image. A color signal box of the appropriate size was displayed as a colored area, where softer was presented as blue, and harder was presented as red. When the cineloop was stable, without dot artifacts, froze it. Elastographic quantitative measurement using a suitable region of interest (ROI) placed in the stiffest region that avoids the cystic component, visible calcifications and the surrounding normal tissue was performed during the investigation. The E_max_, E_mean_, and E_min_ values in the ROI were recorded as kPa for the lesions. A second ROI of the appropriate size was placed in the normal thyroid parenchyma. The elastic ratio (ER) of the mean stiffness for the lesion-to-normal parenchyma were calculated. Repeating the process, at least 3 successive measurements were carried out for each nodule to choose the best SWE image at our institution. If the nodule was too large to scan, multiple measurements for different regions were adopted.

### Cytological and histopathological features

All of the nodules (Figure 1) underwent US-guided FNA by the one of 7 radiologists who performed conventional US and SWE on the same day. FNA biopsy was applied to completely or partially solid nodules, only colloid cysts and so-called spongiform nodules would be excluded. US-guided FNA was performed by using a 23-gauge needle without local anesthesia [25] and a 2mL disposable plastic syringe with a freehand technique. At least three slides were obtained for cytological analysis. All of the slides containing FNA results were analyzed by an expert cytopathologist using the six-tiered diagnostic Bethesda system [26].

All of 313 nodules had follow-up US-guided FNA or thyroid surgery after cytologic diagnosis results. The final benign or malignant results of follow-up US-guided FNA or thyroid surgery was used as the gold standard in this study.

### Statistical analysis

There are two cases involving the combined application of SWE and TI-RADS. (1) In one case, when one or both methods resulted in positivity, the result was considered positive. Only when both methods resulted in negativity, the result was considered negative. This relationship between both methods is then termed “parallel”. (2) In the other case, only when both methods resulted in positivity, the result was considered as positive. When one or both methods were negative, the result was considered negative. The relationship between both is then termed “tandem”. The positive results of SWE and TI-RADS respectively recorded as “1”, and the negative results of two methods for “0”. When both of methods were “1” simultaneously, both forms of “parallel” and “tandem” were recorded as “1”. When both of methods were “0” simultaneously, both forms of “parallel” and “tandem” were recorded as “0”. When one of two methods was “1” alternatively, the result of “parallel” was recorded as “1” and the result of “tandem” for “0”. Organizing these data, and using the results of cytology and pathology as a diagnostic criterion, we then calculated the diagnostic performance of “parallel” and “tandem”.

Descriptive statistics were applied to all of the collected variables expressed as frequency tables for categorical data or mean values ± standard deviations for continuous data. The SWE values and TI-RADS scores of all lesions were correlated with the cytological or pathologic diagnosis of nodules. Student's *t* test was used to assess the differences between two groups of quantitative variables. If the variance of the quantitative variables was unequal, Welch's correction was used. Links between two qualitative variables were estimated using Chi-squared test or Fisher's exact test. The diagnostic performance of SWE elasticity indices (EIs) and TI-RADS scores was assessed by analyzing receiver operating characteristic (ROC) curves for predicting malignancy, and optimal SWE cut-off values and TI-RADS scores yielding the maximal sum of sensitivity, specificity, positive likelihood ratio (PLR), negative likelihood ratio (NLR), Youden's index, positive predictive value (PPV), negative predictive value (NPV), areas under the ROC curve (AUC) were calculated.

SPSS 19.0 statistic software was applied to all of the statistical analyses in our study. For all of the analyses, a *P* value less than 0.05 was considered statistically significant.
